# Portioning-Out and Individuation in Mandarin Non-interrogative *wh*-Pronominal Phrases: Experimental Evidence From Child Mandarin

**DOI:** 10.3389/fpsyg.2020.592281

**Published:** 2021-02-16

**Authors:** Aijun Huang, Francesco-Alessio Ursini, Luisa Meroni

**Affiliations:** ^1^Department of English, School of Foreign Languages, Shanghai Jiao Tong University, Shanghai, China; ^2^School of Chinese Language and Literature, Central China Normal University, Wuhan, China; ^3^Utrecht Institute of Linguistics OTS, Utrecht University, Utrecht, Netherlands

**Keywords:** countability, individuation, Mandarin, classifiers, *wh*-pronominal phrases, portioning-out

## Abstract

Portioning-out and individuation are two important semantic properties for the characterization of countability. In Mandarin, nouns are not marked with count-mass syntax, and it is controversial whether individuation is encoded in classifiers or in nouns. In the present study, we investigates the interpretation of a minimal pair of non-interrogative *wh*-pronominal phrases, including *duo-shao*-N and *duo-shao-ge*-N. Due to the presence/absence of the individual classifier *ge*, these two *wh*-pronominal phrases differ in how they encode portioning-out and individuation. In two experiments, we used a Truth Value Judgment Task to examine the interpretation of these two *wh*-pronominal phrases by Mandarin-speaking adults and 4-to-6-year-old children. We found that both adults and children are sensitive to their interpretative differences with respect to the portioning-out and individuation properties. They assign either count or mass readings to the bare *wh*-pronominal phrase *duo-shao*-N depending on specific contexts, but only count readings to the classifier-bearing *wh*-pronominal phrase *duo-shao-ge*-N. Moreover, the portioning-out and individuation properties associated with the individual classifier *ge* emerge independently in the course of language development, with the portioning-out property taking precedence over the individuation property. Taken together, the present study provides new evidence for the view that the portioning-out and individuation properties in Mandarin are encoded in classifiers rather than in nouns, and these two semantic properties are two distinct components in our grammar.

## Introduction

Since Jespersen (1924, p. 198), countability of nominal expressions is usually defined as the property of “portioning out” (Borer, 2005) and individuating referents. Thus, portioning-out and individuation are two core concepts involved in the characterization of countability (Quine, 1960; McCawley, 1975; Pelletier, 1979, 2012; Ware, 1979; Allan, 1980; Gordon, 1982, 1985; Macnamara, 1982; Bloom, 1990; Chierchia, 1998, 2010; Barner and Snedeker, 2005, 2006; Borer, 2005; Bale and Barner, 2009; Rothstein, 2010, 2017; among many others). For the sake of clarity, we briefly introduce our use of these two concepts, while giving a more detailed account in Sections “Portioning-Out and Individuation in Mandarin” and “Portioning-Out and Individuation in Mandarin *wh*-Pronominal Phrases.”

We define the portioning-out function of a linguistic element as the process of carving out a discrete unit for counting (cf. Au Yeung, 2005; Borer, 2005; Rullmann and You, 2006; Huang, 2009; Huang and Lee, 2009; Li, 2013; Zhang, 2013). Two related semantic dimensions are involved in the concept of portioning-out: cardinality (singularity/plurality) and discrete units of counting^1^. To illustrate, the classifier *kuai* in sentence (1) specifies one chunk of entity, in which the cardinality is one and the discrete unit of counting is ‘chunk.’ So, the classifier phrase *kuai-pinggo* ‘CL_*kuai*_-apple’ refers to an apple chunk. By contrast, in the absence of a classifier, bare nouns in Mandarin are underspecified in quantity and no unit of counting is specified. So the bare noun *pingguo* in (2) can denote one or more individual apple(s)/apple chunk(s), and even apple substance in form of pureé.

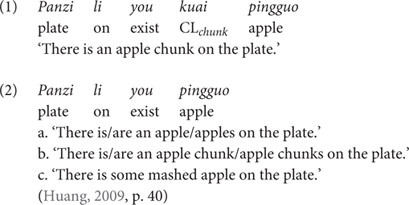

On the other hand, the individuation function of a linguistic element is a more restrictive notion, since it is defined on the basis of the portioning-out function. To illustrate, similar to the classifier *kuai* ‘chunk’ introduced above, the individual classifier *ge* in (3) specifies a discrete unit of counting. However, unlike *kuai*, the discrete unit of counting associated with *ge* has to be a kind of discrete units that corresponds to the natural unit of individual objects. Thus, the individuation function of individual classifiers in Mandarin requires that their associated nouns must denote individual objects, and cannot be a non-individual entity such as apple substance or an apple chunk. Taken together, due to the portioning-out and individuation functions of *ge*, the classifier phrase *ge-pingguo* in (3) denotes an individual, ‘whole’ apple. The identification of the individuation function of individual classifiers distinguishes this type of classifiers from non-individual classifiers, a fact that is well-acknowledged in the literature (e.g., Chao, 1968; Cheng and Sybesma, 1998, 1999).

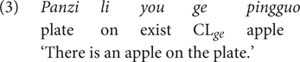

Typologically distinct languages, as defined here by the presence/absence of the count-mass syntax (e.g., English versus Mandarin), generally differ in their ways of portioning-out and individuating referents. However, there is a fundamental issue that linguists and psycholinguists have been pursuing, namely, whether or not the apparent cross-linguistic distinctions reveal some language universals in expressing and representing countability. The present study attempts to address this issue by investigating how portioning-out and individuation in Mandarin are encoded and acquired in the child grammar.

Consider English first. In this language, plural morphology and determiners portion out and individuate referents of associated nominal expressions (Borer, 2005). For example, in (4) and (5), due to the presence/absence of the plural marker *-s*, the count and mass uses of the same noun *chicken* in the phrases ‘*the chickens*’ and ‘*the chicken*’ differ in portioning out and individuation (Borer, 2005). While *the chickens* denotes a multiple number of individual chickens without specifying how big these chickens are, *the chicken* denotes a certain amount of chicken mass without specifying whether there exist individual chickens (cf. Bale and Barner, 2009).

(4) He did not eat the chickens this evening.
(5) He did not eat the chicken this evening.

Previous empirical research shows that English-speaking children acquire the portioning-out function of count determiners and plural morphology earlier than their individuation function. In particular, the early acquisition of the portioning-out function can be found in the study of Kouider et al. (2006). In this study based on a Preferential Looking Task, English-speaking Children as young as 36 months responded properly toward the distinct portioning-out information expressed by singular–plural markers. When these young children were presented with the sentences like *look at the blickets* (in which the plural marker *–s* was attached to the novel word *blicket*), they looked at the set of multiple individual objects. By contrast, when they were presented with the sentences like *look at the blicket* (in which the singular form of *blicket* was used), they looked at a single individual object. This suggests that children are aware of the plural units of portioning-out as encoded by *–s*, as distinguished from the singular unit of portioning-out encoded by the same word without the plural marker (see also Barner and Snedeker, 2005, 2006).

In contrast with the early emergence of the portioning-out function of English count determiners and plural morphology, the individuation function is delayed in English-speaking children. The delay of the individuation function is manifested by children’s comprehension and production of English number words, count determiners (i.e., *a*, *more*, *every*, and *both*) and plural morphology. Unlike adults, 3-to-4-year-old children treat discrete physical objects (i.e., parts of broken individual objects) as units of portioning-out. This kind of non-adult-like behavior is reported in Brooks et al. (2011) (see also Shipley and Shepperson, 1990; Wagner and Carey, 2003). For example, on a Counting Task in which children were asked with questions like ‘*can you count the shoes*?,’ children included in their counting partial objects (e.g., three divided parts of a shoe), as well as whole objects. This kind of non-adult-like response can be attributed to the delay of the individuation function of the plural morphology in 3-to-4-year-old English-speaking children, in the sense that children have not acquired the linguistic knowledge that the multiple entities associated with the plural morphology must be individuals.

Unlike English, classifiers languages like Mandarin do not have grammatical categories like plural morphology and determiners to encode portioning-out and individuation. Rather, classifiers are used to express the meanings associated with portioning-out and individuation (Borer, 2005; Li, 2013; Zhang, 2013). In addition, Mandarin has another peculiar typological feature that is not attested in English, i.e., the use of bare nouns. Unlike English in which nouns are used either in a count or a mass form, nouns in Mandarin can appear in a bare form, in which no grammatical category is used to mark their countability. These typological features of Mandarin nouns and classifiers bring about heated discussion and debates regarding the expression and representation of countability in this language. In particular, it is widely accepted that Mandarin classifiers encode portioning-out (Chen, 2003; Au Yeung, 2005; Borer, 2005; Huang, 2009; Huang and Lee, 2009; Li, 2013; Zhang, 2013). However, it is controversial with regard to the encoding of individuation. While some scholars contend that individuation is encoded **in** nouns (Chao, 1968; Fung, 1993; Doetjes, 1997; Cheng and Sybesma, 1998, 1999, 2005; Cheng et al., 2008; Liu, 2014), others argue that individuation is encoded and specified by Mandarin individual classifiers (Hansen, 1983; Bach, 1989; Graham, 1989; Krifka, 1995; Chierchia, 1998; Borer, 2005; Huang, 2009; Huang and Lee, 2009; Rothstein, 2010; Pelletier, 2012).

To address the theoretical controversy, the present study investigates portioning-out and individuation associated with bare nouns and classifiers co-occurring with non-interrogative Mandarin *wh*-pronominal phrases, a new area of research that has barely drawn linguists’ attention so far. We focus on a minimal pair of *wh*-pronominal phrases when they are used in conditionals (e.g., Cheng and Huang, 1996; Lin, 1996), namely, the bare *wh*-pronominal phrase ‘*duo-shao* N’ (‘bare’ in the sense that there is no co-occurring classifier) and the classifier-bearing *wh*-pronoun phrase ‘*duo-shao-ge* N’ (in which the individual classifier *ge* appears between the *wh*-pronoun *duo-shao* and the head noun). In two experiments, we used a Truth Value Judgment Task (Crain and Thornton, 1998) to test the interpretation of non-interrogative sentences containing these two *wh*-pronominal phrases by Mandarin-speaking adults and children. Our experimental data provide strong evidence for the view that (i) portioning-out and individuation in Mandarin are encoded in classifiers rather than in nouns; (ii) portioning-out and individuation are two distinct linguistic components in the characterization of countability in Mandarin, with portioning-out taking precedence over individuation (Borer, 2005; Huang, 2009; Huang and Lee, 2009; Duan, 2011). In a word, the present study contributes new data to adjudicate the theories of the count-mass issues in Mandarin. From a cross-linguistic perspective, the similar developmental pattern on the acquisition of portioning-out and individuation between Mandarin and English indicates some language universals in encoding portioning-out and individuation, despite their distinct ways of encoding these two semantic properties.

The remaining parts of the present study are arranged as follows. Sections “Portioning-Out and Individuation in Mandarin” and “Portioning-Out and Individuation in Mandarin *wh*-Pronominal Phrases” introduce portioning-out and individuation in Mandarin and in Mandarin *wh*-pronominal phrases, and Section “Portioning-Out and Individuation in Child Mandarin” introduce how these two semantic functions of Mandarin classifiers are acquired by Mandarin-speaking children. Section “Experiments” reports our two experiments. Section “General Discussion and Conclusion” discusses the experimental data and concludes the paper.

### Portioning-Out and Individuation in Mandarin

Nouns in Mandarin are not systematically marked with the count-mass syntax like some Indo-European languages such as English do, and Mandarin nouns can be used in bare forms. On the other hand, the expression of countability is closely related to the Mandarin classifier system (e.g., Krifka, 1995; Cheng and Sybesma, 1998, 1999; Borer, 2005). These typological features of Mandarin nouns and classifiers generate heated discussion and debates regarding the expression and representation of portioning-out and individuation in this language.

As introduced in Section “Introduction,” the portioning-out function of Mandarin classifiers carves out a unit for counting. This function is evident if one looks at the interpretive differences between minimal pairs of bare nouns and classifier phrases. We have seen that while bare nouns are not portioned out and thus are underspecified in quantification, a classifier-noun phrase specifies a discrete unit of counting for the interpretation of associated nouns. The portioning-out function of classifiers as it is used here is identified via various terms in traditional Chinese grammar, e.g., *danwei ci* ‘unit word’ (Lü, 1942), *danwei mingci* ‘unit-nominal’ (Wang, 1944), *shuwei ci* ‘counting-unit word’ (Gao, 1948).

The portioning-out function is a basic function attested in all Mandarin classifiers, in the sense that each and every type of Mandarin classifiers specifies a discrete unit of counting (Greenberg, 1972, p. 26; Krifka, 1995; Au Yeung, 2005; Huang, 2009; Huang and Lee, 2009; Zhang, 2013, pp. 36–38). Importantly, the unit of counting does not specify the weight or size of the quantified entities^2^. Hence, the CL-N phrase *ge pingguo* in (3) above does not indicate whether the individual apple is big or small (cf. Barner and Snedeker, 2005, 2006; Bale and Barner, 2009). This concept is important for our experimental design, as it will become clear later.

The encoding of individuation in Mandarin is a controversial topic in the literature. Some scholars argue that individuation is encoded in nouns, and Mandarin nouns are divided into count nouns and mass nouns based on their denotation (Doetjes, 1997; Cheng and Sybesma, 1998, 1999, 2005; Cheng et al., 2008; Liu, 2014). Count nouns refer to nouns that denote entities that “present themselves naturally in discrete, countable units” (Cheng and Sybesma, 1998, p. 385), such as *ping-guo* ‘apple.’ On the other hand, mass nouns refer to nouns like *shui* ‘water’ whose denotation does not present itself naturally in discrete entities. As for the function of Mandarin classifiers, Cheng and Sybesma (1998, 1999) propose that individual classifiers (or ‘count classifiers’ in their terminology) “name” inherent units of counting that are encoded in associated count nouns, or “make the semantic partitioning of count nouns syntactically visible” (p. 520) (cf. Doetjes, 1997). On the other hand, other types of classifiers (or ‘massifiers’ in their terminology) “create” units of counting. The distinction between count classifiers and massifiers is regarded as the realization of the grammatical count-mass distinction at the classifier level in Mandarin [cf. see Tang, 2005; Li, 2013; Zhang, 2013 for their arguments against Cheng and Sybesma (1998, 1999) account]. The account proposed by Cheng and Sybesma is named as the ‘lexico-syntactic approach’ by Lin and Schaeffer (2018).

Differing from the lexico-syntactic approach, some other scholars contend that bare nouns in Mandarin do not specify their count or mass status, and it is classifiers that determine and specify individuation of a noun (Hansen, 1983; Bach, 1989; Graham, 1989; Krifka, 1995; Chierchia, 1998; Borer, 2005; Huang, 2009; Huang and Lee, 2009; Rothstein, 2010; Pelletier, 2012). We focus on the accounts proposed by Borer (2005) and Pelletier (2012) here. Both accounts argue that classifiers determine individuation in Mandarin, but they differ in their characterization of bare nouns, as detailed below.

In Borer’s (2005) account, both count nouns and mass nouns are grammatically constructed. Thus, “all nouns, in all languages, are mass, and are in need of being portioned out, in some sense, before they can interact with the ‘count’ system (p. 93).” From a cross-linguistic perspective, Borer proposes that the portioning-out function is accomplished in Mandarin through the projection of count classifiers, on a par with the portioning-out function of plural morphology and count determiners and quantifiers in English. In this account, bare nouns in Mandarin, in the absence of a portioning-out category, are regarded as having only their default mass interpretation.

In Pelletier’s (2012) account, the count-mass interpretation involves the interaction between four features at two levels: +COUNT*_*syn*_* and +MASS*_*syn*_* at the syntactic level, and +COUNT*_*sem*_* and +MASS*_*sem*_* at the semantic level. In particular, at the semantic level, “the semantic value of every lexical noun contains all the values of which the noun is true (p. 19).” Thus, both count and mass values are available, and nouns are unspecified in the lexicon for their count and mass interpretation before they enter the syntax. When the syntactic feature +COUNT*_*syn*_* is introduced, the opposite semantic feature +MASS*_*sem*_* on the noun is deleted, resulting in a count interpretation. The mass interpretation is obtained in a similar way by introducing the syntactic feature +MASS*_*syn*_* and deleting the semantic feature +COUNT*_*sem*_*. Under Pelletier (2012) account, number-marking languages introduce the feature +COUNT*_*syn*_* via plural morphology and in combination with count determiners, and introduce +MASS*_*syn*_* in combination with other determiners. Classifier languages, on the other hand, introduce the +COUNT*_*syn*_* or +MASS*_*syn*_* in construction with count and mass classifiers, respectively. When it comes to the interpretation of nouns in Mandarin, since neither +COUNT*_*sem*_* nor +MASS*_*sem*_* is deleted, bare nouns in Mandarin are flexible with count and mass readings. When co-occurring with a count classifier, nouns allow only the individual-denoting reading.

Overall, portioning-out and individuation are two fundamental notions related to the count-mass interpretation of nominal expressions in Mandarin. Scholars agree that Mandarin classifiers specify a discrete unit for counting and thus encode the portioning-out function. However, it is still controversial whether individuation in Mandarin is encoded and specified in nouns or in classifiers, and how to interpret bare nouns. In the present study, we investigate portioning-out and individuation in another under-investigated area, namely the Mandarin *wh*-pronominal system and then discuss, after presenting our experimental data, which account fares better to characterize countability of Mandarin.

### Portioning-Out and Individuation in Mandarin *wh*-Pronominal Phrases

To investigate portioning-out and individuation in the Mandarin *wh*-pronominal system, we focus on two *wh*-pronominal phrases, i.e., *duo-shao*-N and *duo-shao-ge*-N. The difference between these two *wh*-pronominal phrases is that while *duo-shao*-N is ‘bare,’ in the sense that it includes no classifier, *duo-shao-ge*-N includes the individual classifier *ge* in its lexical morphology^3^. Next we show that, the interpretive differences between *duo-shao* and *duo-shao-ge*-N are in parallel with those between bare nouns and CL_*ge*_-N as shown in examples in (1) and (3).

Consider sentences in (6)–(7), in which *duo-shao*-N and *duo-shao-ge*-N occur in conditional structures, a typical structure licensing the non-interrogative use of *wh*-pronouns (e.g., Cheng and Huang, 1996; Lin, 1996; Chierchia, 2000; Liu, 2016). These are the two types of sentences we used in our experiments, as will be shown later.

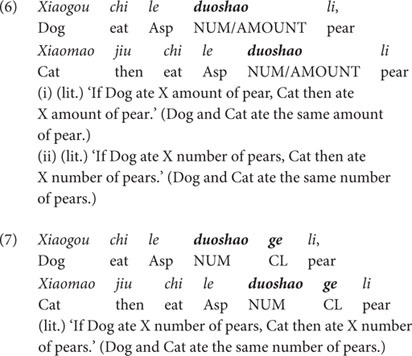

*Duo-shao*-N in (6) and *duo-shao-ge*-N in (7) receive distinct semantic interpretations in portioning-out and individuation. In (6), the bare *wh*-pronoun phrase *duo-shao*-*li* does not contain a linguistic element encoding the portioning-out and individuation functions. Therefore, this phrase is underspecified in terms of the discrete unit of counting, and the referents of the associated noun *li* ‘pear’ can be measured by multiple scales, such as a cardinal scale, a scale of weight, a scale of volume, etc. This explains why sentence (6) is ambiguous. On a substance-denoting reading, this sentence states that Dog and Cat ate the *SAME AMOUNT* of pear(s), in which the referent of *li* ‘pear’ is measured on a scale of weight and other information such as the number and the shape of pear(s) is not specified. Alternatively, on an individual-denoting reading, this sentence means that Dog and Cat ate the *SAME NUMBER* of pears, in which the referent of *li* ‘pear’ is measured on a cardinal scale and information such as the size or weight of the pears is not specified. These are two possible readings that can be conveyed by sentence (6), among many other possible readings. These are also the readings we aim to trigger for the interpretation of the bare *duo-shao*-N in Experiment 1.

By contrast, in interpreting *duo-shao-ge*-*li* phrases in sentence (7), a discrete unit of counting is specified, due to the portioning-out function of *ge*. Furthermore, the individuation function of *ge* requires that this discrete unit corresponds to the natural unit of individual objects as denoted by the associated noun. Thus, *duo-shao-ge*-*li* in sentence (7) must denote individual pears and this sentence can only have the individual-denoting reading: Dog and Cat ate the *SAME NUMBER* of pears. The portioning-out function of *ge* is examined in Experiment 1, and its individuation function of *ge* is examined in Experiment 2.

Overall, the interpretation of the sentences in (6)–(7), together with the interpretation of (1)–(3) in Section “Introduction,” boils down to one parameter of variation: the presence/absence of the individual classifier *ge* determines their portioning-out and individuation. In the absence of such an individual classifier, bare nouns and *duo-shao*-N are underspecified on portioning-out and individuation, thus allowing both count readings (i.e., the ‘individual-denoting’ reading) and mass readings (i.e., the ‘substance-denoting’ reading). By contrast, the presence of the individual classifier *ge* in classifier-bearing nominal phrases and *duo-shao-ge*-N determines that they can only convey the count readings (i.e., the ‘individual-denoting’ reading). Next, we will see how these two semantic functions are acquired by Mandarin-speaking children. We will first review some previous studies, then report our own experiments.

### Portioning-Out and Individuation in Child Mandarin

It has been reported that the portioning-out and individuation functions of Mandarin classifiers develop independently in the course of language development. In particular, Huang (2009), Huang and Lee (2009), and Duan (2011) report that the portioning-out function emerges earlier than the individuation function in Mandarin-speaking children’s interpretation of Mandarin classifiers. Differing from previous research, which investigates either the interpretation of classifiers (Chien et al., 2003; Li et al., 2008, 2010; Cheung et al., 2010) or bare nouns (Lin and Schaeffer, 2018), Huang and Lee and Duan examine both the interpretation of bare nouns and classifier-bearing structures. Let us see the details below.

**FIGURE 1:**
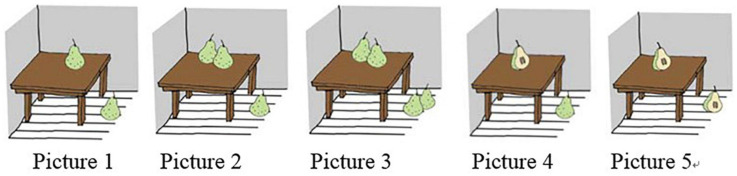
Picture items from Huang, 2009 (p. 98).

Huang (2009) and Huang and Lee (2009) investigated the interpretation of the sentences containing three individual classifiers, *ge*, *tiao* and *zhang*, as compared with the interpretation of sentences containing bare nouns. According to them, the portioning-out function of these three individual classifiers is acquired by children as young as 3 years old, while their individuation function is not acquired until they reach 6 years of age. These two functions were tested using a picture verification task with 3-to-6-year-old children. Children were presented with minimal pair sentences which differ only in the presence or absence of an individual classifier, as exemplified in (8) (with CL-N *ge lizi* ‘CL-pear) and (9) (with the bare noun *lizi* ‘pear’). Each of these two sentences was tested with five pictures as shown in Figure 1. Among the pictures, Pictures 1–3 were used to test the portioning-out function of *ge* and Pictures 4–5 were used to test its individuation function.

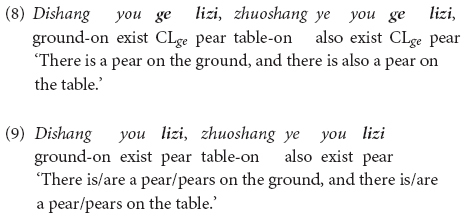

In their response to the individual classifier-bearing sentence in (8), children as young as 3 years correctly accepted the sentence as a correct description of Picture 1 in Figure 1 (which shows one object on the table/on the floor for the interpretation of the structure *ge lizi* ‘CL-pear’), but rejected the sentence for Picture 2 and Picture 3 (which contain more than one object on the table for the interpretation of *ge lizi*). By contrast, in their response to the bare noun-bearing sentence in (9), these children accepted the sentence for all of the three pictures (Pictures 1–3). Based on children’s responses, these two studies concluded that while young children are aware that a singular unit of counting is involved in individual classifier-bearing sentences as in (8), but not necessarily in bare noun-bearing sentences as in (9).

Moreover, when tested for the individuation function of individual classifiers, 3-to-5-year-old children judged Picture 4 and Picture 5 in Figure 1 (which contain partial objects) to be good descriptions of both the sentence (8) (with an individual classifier) and the sentence (9) (with a bare noun), while 6-year-old children started being adult-like and thus only accepted these two pictures with the bare noun sentence (9) but not with the individual classifier-bearing sentence (8). The younger children’s non-adult-like behavior is attributed by the authors to the lack of the individuation function of individual classifiers in the early stage of language development, such that children of younger age do not know that individual classifier structures must refer to individual whole objects.

Duan (2011) looked into the acquisition of collective classifiers. Collective classifiers in Mandarin encode the portioning-out and individuation functions, specifying that the associated nouns denote multiple individual objects (cf. Huang, 2009; Zhang, 2013). Duan reported that 6-to-10-year-old children exhibit adult-like responses when tested with the portioning-out function of collective classifiers, but their individuation function is not acquired until children reach 10 years of age. She tested five collective classifiers, including *shuang* ‘pair,’ *dui* ‘pair,’ *qun* ‘group,’ *chuan* ‘string’ and *pai* ‘row.’ To illustrate, we use *dui* ‘pair’ here. When it comes to the portioning-out function of this collective classifier, children correctly accepted sentences like (10) in situations presenting a pair of objects, but rejected the same sentences in situations presenting one single object, three objects, or two pairs of objects (Experiment 1).

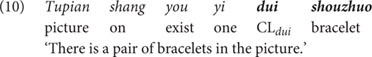

As for the individuation function associated with *dui, dui*-containing sentences were judged against three different pictures, one with two whole objects, one with two partial objects of the same shape, and one with two partial objects of different shapes (Experiment 5). Her findings indicate a developmental pattern. For the 6-year-old group, in addition to their acceptance of the sentences for the whole-object pictures, a large percentage of children accepted the sentences when presented with the two kinds of partial-object pictures: 75% (for the pictures of partial objects of the same shape); 44% (for the pictures of partial objects of different shapes). To compare, in the 8-year-olds and 10-year-olds, the percentage for allowing the test sentences to match the two kinds of partial object situations dropped to 30% or so, close to the adult level.

Summing up, previous research shows that the portioning-out function of Mandarin classifiers is acquired by children as young as 3 years old, while the individuation function is not acquired until they reach 6 years of age. Therefore, these two functions develop independently in the course of language development, with the portioning-out function being acquired earlier than the individuation function. The empirical data also show that bare nouns are underspecified in portioning-out and individuation, allowing both count and mass readings. Now we turn to our experiments.

## Experiments

In what follows we will present two experiments we conducted in order to investigate the acquisition of portioning-out and individuation involved in the comprehension of Mandarin *wh*-pronominal phrases with and without the individual classifier *ge*: *duo-shao*-N and *duo-shao-ge*-N. In particular, Experiment 1 focuses on the portioning-out function of *ge* and the interpretation of bare nouns, and Experiment 2 on the individuation function of *ge*.

### Experiment 1

Experiment 1 investigated the acquisition of portioning-out as involved in the interpretation of *duo-shao*-N and *duo-shao-ge*-N. The experimental design is as follows.

#### Test Sentences, Research Questions and Predictions

There are two types of test sentences, exhibiting the non-interrogative uses of *duo-shao*-N and *duo-shao-ge*-N in the conditional structure. Recall that this structure requires that the pair of *wh*-pronouns in the antecedent and in the consequent denotes the same quantificational information, as exemplified in (6) and (7), repeated here as (11) and (12).

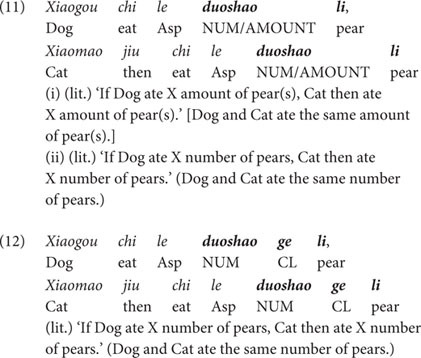

As discussed earlier, due to the absence of a linguistic element encoding the portioning-out function and the individuation function, the bare *duo-shao* sentence in (11) is ambiguous and the referent of the *duo-shao-li* phrase in this sentence can be measured by multiple scales. Among many other possible readings, this sentence can denote an individual-denoting reading (‘Dog and Cat ate the same number of pears’) and a substance-denoting reading [‘Dog and Cat ate the same amount of pear(s)’] when the context highlights an appropriate scale of measurement. To be more specific, the individual-denoting reading can be triggered when a cardinal scale is under consideration, and the substance-denoting reading can be triggered when a scale of weight is in focus. Our experiment will provide appropriate contexts to trigger these two readings, as will be shown shortly.

By contrast, due to the portioning-out function of the individual classifier *ge*, the *duo-shao-ge-li* in (12) specifies a discrete unit of counting. Furthermore, the individuation function of *ge* requires that, this discrete unit of counting corresponds to the inherent natural unit of individual pears as denoted by the noun *li* ‘pear.’ In this case, the referents of the *duo-shao-ge-li* phrase can only be measured by a cardinal scale. Taken together, sentence (12) conveys only an individual-denoting reading (‘Dog and Cat ate the same number of pears’).

It is worthwhile to point out that the individual-denoting reading assigned to *duo-shao-ge-li* in (12) comes from a different source, as compared to the same individual-denoting reading assigned to the bare *duo-shao-li* in (11). As we stated above, the individual-denoting reading in (11) is triggered by context (via a cardinal scale). However, the individual-denoting reading in sentence (12) is imposed by morpho-syntax, i.e., the presence of the individual classifier *ge*. This morpho-syntactic-driven reading cannot be overridden by the context. Thus, we expect that whatever context is provided, sentence (12) only has the individual-denoting reading. We will confirm this in our experiment.

Another important thing we need to clarify is that, even though the individual classifier *ge* in *duo-shao-ge*-N phrases has both the portioning-out and individuation functions, Experiment 1 only tested the portioning-out function. We thus left the examination of the individuation function to Experiment 2. To examine the portioning-out function, we compare the interpretive differences between *duo-shao-ge*-N and *duo-shao-*N in portioning-out: while *duo-shao-*N allows multiple scales of measurement, *duo-shao-ge*-N specifies a discrete unit of counting. As we will show later, in the experiment we provide two different scales of measurements: a cardinal scale and a scale of weight. If participants allow both of these scales of measurement in their interpretation of the bare *duo-shao-*N sentences but only a cardinal scale for the *duo-shao-ge*-N sentences, we can conclude that they are aware of the portioning-out function of *ge*.

Since morpho-syntax (i.e., the presence/absence of a classifier) and contextual information affect the interpretation of these two *wh*-pronominal phrases in specifying a unit of portioning-out, we formulated the following two research questions for Experiment 1. First, we ask whether Mandarin-speaking children will behave like adults and allow both individual-denoting and substance-denoting readings in interpreting bare *duo-shao* sentences, but only individual-denoting readings in interpreting classifier-bearing *duo-shao-ge* sentences. If so, we ask further whether Mandarin-speaking children know that contextual manipulation affects the interpretation of bare *duo-shao* sentences but not that of classifier-bearing *duo-shao-ge* sentences. We predict that the answers to these two questions are positive, considering the early acquisition of the portioning-out function of Mandarin classifiers as reviewed earlier (Huang, 2009; Huang and Lee, 2009; Duan, 2011).

#### Participants

We recruited 20 4-to-5-year-old Mandarin-speaking children from a kindergarten affiliated to Soochow University, Jiangsu Province, China. The child group ranged in age from 4;3.28 to 5;7.13, with a mean age of 4;11.26. Based on previous research on the acquisition of Mandarin classifiers and *wh*-pronouns (Li and Tang, 1991; Huang, 2009; Huang and Lee, 2009; Fan, 2012; Zhou et al., 2012), we estimate this is the youngest age we can test for the portioning-out function of Mandarin classifiers associated with the two *wh*-pronouns. We also included a control group of twenty adults. The adult participants were postgraduate students from Soochow University.

#### Procedures

The experiment used a Truth Value Judgment Task (Crain and Mckee, 1985; Crain and Thornton, 1998). The task involves two experimenters. One experimenter narrates the stories using toys and props. The other experimenter plays the role of a puppet who watches the story alongside the child. At the end of each story, the puppet is invited to explain to the child what has happened in the story. The child’s task is to judge whether the puppet says the right thing or not. If the child informs the puppet that s/he is wrong, then s/he is asked to explain “what really happened?”

The child participants were introduced to the task and tested individually. Each child was given one practice trial to familiarize with the task. Only those children who responded correctly in the practice trial proceeded to the test session. The adult participants were tested on the same stories, but they were tested in a group. After listening to the narration of the stories by the experimenter, the adults were asked to indicate on an answer sheet whether the puppet was right or wrong. As with the child participants, the adult participants were asked to provide a justification if they judged that the puppet had offered an inaccurate description of the story. They were told to work on their sheet independently and were not allowed to discuss among themselves. The practice trial was also given to the adult participants at the beginning of the testing.

#### Test Conditions

There are two test conditions, representing two distinct contexts that are associated with portioning-out: the number of individual objects and the amount/size of entities (cf. Barner and Snedeker, 2005, 2006; Bale and Barner, 2009). Test Condition 1 is designed to create an amount-oriented context, by comparing the *AMOUNT* of two entities which differ in size (e.g., eating two big pumpkins versus eating two small pumpkins). Thus, a scale of weight is embedded in the design of this condition. Test Condition 2 is designed to create an individual-oriented context, highlighting the *EQUAL NUMBER* of entities acted upon by two animal characters (e.g., each of the animals makes two flowers, and all the flowers are considered as good art works, regardless of their size). Clearly, a cardinal scale is designed in this condition.

Each of the two types of test sentences shown in (11) and (12) was tested in the two distinct contexts. We expect that the sentences containing the bare *wh*-pronoun *duo-shao* [e.g., sentence in (11)] are ambiguous, and aim to trigger two distinct readings upon our contextual manipulation: a substance-denoting reading in the amount-oriented context and an individual-denoting reading in the individual-oriented context. On the other hand, we expect that the sentences containing the classifier-bearing *wh*-pronoun *duo-shao-ge* [e.g., sentence in (12)] will select only the individual-denoting reading, no matter how the context is manipulated. The experimental design of Experiment 1 is summarized in Table 1 below.

**TABLE 1:** The experimental design of Experiment 1.

**Morpho-syntactic factor**		**Contextual information**	
		**Amount-oriented context (Condition 1)**	**Individual-oriented context (Condition 2)**
Absence of the individual CL *ge*	*Duo-shao*	Substance-denoting reading (‘No’ responses)	Individual-denoting reading (‘Yes’ responses)
Presence of the individual CL *ge*	*Duo-shao-ge*	Individual-denoting reading (‘Yes’ responses)	Individual-denoting reading (‘Yes’ responses)

#### Test Materials

From Table 1, we can see that two independent variables are created in the experimental design, namely the morpho-syntactic factor (i.e., presence/absence of the individual classifier *ge*) and contextual information (i.e., amount-oriented context versus individual-oriented context). Thus, Experiment 1 is designed to investigate how these two factors determine and influence the portioning-out associated with the *wh*-pronouns *duo-shao* and *duo-shao-ge*. We will now illustrate the experimental design with some typical trials.

**FIGURE 2:**
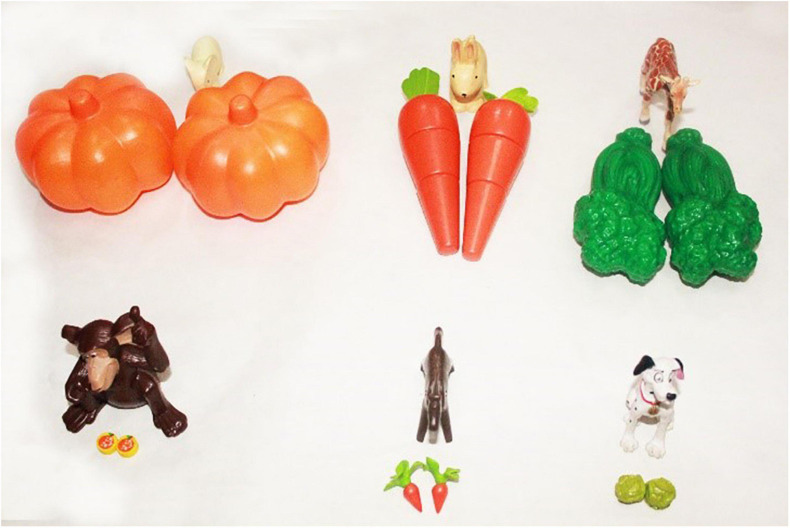
Last scene of the story (the amount-oriented context).

First, let us consider the amount-oriented context, as shown in (13). In this story, there were six animal characters eating three kinds of vegetables. Among these six animals, three animals each ate two big vegetables and became very full, while the other three animals each ate two small vegetables of the same kind and were still hungry. With this design, these six animals constituted three pairs, with each pair eating two vegetables of the same kind, but of a different size (i.e., Elephant eating two big pumpkins versus Monkey eating two small pumpkins; Rabbit eating two big carrots versus Horse eating two small carrots; Giraffe eating two big cabbages versus Dog eating two small cabbages). Importantly, the uneven amount of food eaten by each pair of the animal characters is significant, as the big amount made one animal full, while the small amount did not relieve the other animal’s hunger at all. The last scenario of the story is shown in the picture in Figure 2.

(13) Story for the amount-oriented context (Condition 1).
Rabbit, Elephant, Giraffe, Horse, Monkey, and Dog went to buy vegetables. Rabbit, Elephant, and Giraffe each bought two big vegetables: Rabbit bought two big carrots, Elephants two big pumpkins, and Giraffe two big cabbages; they ate all their big vegetables, and became very full. Horse, Monkey, and Dog each bought two small vegetables: Horse bought two small carrots, Monkey two small pumpkins, and Dog two small cabbages. They ate the small vegetables, but were still hungry.

Against this kind of scenarios, both *duo-shao* sentences and *duo-shao-ge* sentences were tested in the same participants. In the case of *duo-shao* sentences, the puppet was asked to use three *duo-shao* sentences to compare the quantity of vegetables eaten by the three pairs of animals. This allowed us to introduce three tokens of the *duo-shao* sentences in a single story. We use the sentence in (14) as an example to illustrate the structure of the test sentences, and the other two sentences are of the same sentence structure. As discussed earlier, the *duo-shao* sentences are ambiguous between the individual-denoting reading (‘X and Y ate the same number of vegetables’) and the substance-denoting reading [‘X and Y ate the same amount of vegetable(s)’]. However, since the amount-oriented context underscores the amount/volume of the vegetables eaten by each pair of the two animal characters, the substance-denoting reading (i.e., ‘X and Y ate the same amount of vegetable(s)’) should be the favored reading in this amount-oriented context, if participants are sensitive to the context. Since this reading did not match the situation in the story [as X and Y in the story ate different amount of vegetable(s)], the test sentences were false descriptions of the story and participants were expected to reject the *duo-shao* sentences in this condition.

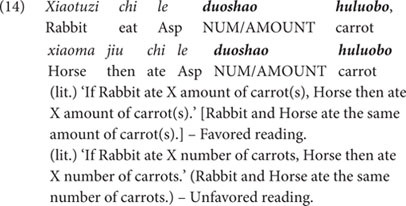

In the same vegetable-eating scenarios as shown in (13), the classifier-bearing *duo-shao-ge* sentences were also presented, as exemplified in (15) below. (Noted that in the actual testing, the *duo-shao-ge* sentences were tested in separate sessions and the animal characters were also changed to different ones. We use the same animal names as in (14) for ease of exposition).

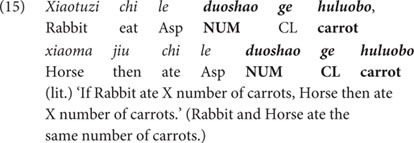

Due to the presence of the individual classifier *ge* in the sentence (15), this sentence allows only the individual-denoting reading: Rabbit and Horse ate the same number of carrots. Obviously, this sentence is a true description of the story, as these two animals did eat the same number of carrots (i.e., two carrots). Thus, we expect that participants would accept the *duo-shao-ge* sentences in this amount-oriented context.

To sum up, in the amount-oriented context, we expected that participants would tend to reject the bare *duo-shao* sentence and assign the substance-denoting reading, but accept the classifier-bearing *duo-shao-ge* sentences and exclusively assign the individual-denoting reading in the same amount-oriented context.

**FIGURE 3:**
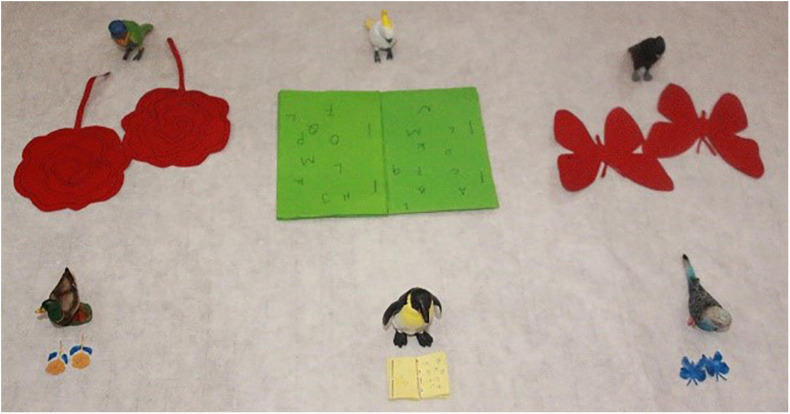
Last scene of the story (individual-oriented context).

Now consider the story designed for the individual-oriented context, as shown in (16). In this story, there were six animal characters doing three kinds of paper crafts: three animals each made two big paper crafts, and the other three animals each made two small paper crafts of the same kinds. Therefore, the six animals constituted three pairs, with each pair making two paper crafts of the same kinds, but of different sizes (Rainbow Bird made two big flowers and Duck made two small flowers; White Bird made two big books and Penguin made two small books; Black Bird made two big butterflies and Blue Bird made two small butterflies). The size difference did not affect the assessment of the animals’ work, as all of the paper crafts were greatly cherished by Fairy. The last scenario of the story is showed in Figure 3.

(16) Story for the individual-oriented context (Condition 2).
Fairy is going to have her birthday. To celebrate her birthday, her friends Rainbow Bird, White Bird, Black Bird, Duck, Penguin and Blue Bird discuss to make some gifts for her. They decide to make three kinds of paper crafts: Rainbow Bird makes two big red flowers and Duck two small orange flowers; White Bird makes two big letter books and Penguin two small number books; Black Bird makes two big red butterflies and Blue Bird two small blue butterflies. Fairy likes all of the paper crafts made by her friends, and kisses each of them.

As we did in the amount-oriented context, both *duo-shao* sentences and *duo-shao-ge* sentences were used in this individual-oriented context to compare the performance of the three pairs of animals. In the case of *duo-shao* sentences, the puppet produced three *duo-shao* sentences at the end of the story to each participant. An example sentence is given in (17) for illustration. In this individual-oriented context, the individual-denoting reading (i.e., ‘X and Y made the same number of paper crafts’) should be preferred, even though the *duo-shao* sentence is ambiguous. This reading matched the story situation (as X and Y did make the same number of paper crafts), so these three *duo-shao* sentences should be accepted as three true descriptions of the story.

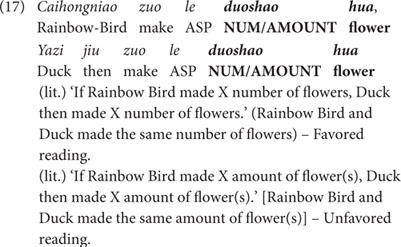

In the same craft-making scenarios as shown in (16), the classifier-bearing *duo-shao-ge* sentences were also presented, as exemplified in (18) below. (Again, in the actual testing, the *duo-shao-ge* sentences were tested in separate sessions and the animal characters were changed to different ones. We use the same animal names as in (17) for ease of exposition).

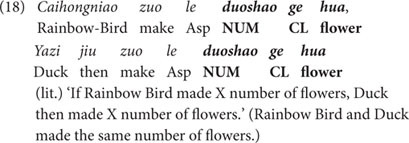

In addition to the test sentences, the puppet also produced a filler sentence before or after each test sentence. The filler sentences were true or false. They served to obscure the research purpose of the study, and to ensure that children remained aware of the task.

To wrap up, we designed a vegetable-eating story and a paper craft-making story for bare *duo-shao* sentences, and two similar stories (i.e., only a change of animal characters) for the individual classifier-bearing *duo-shao-ge* sentences. Overall, we had four stories in total in this experiment. We adopted the within-subject design, testing each participant with the two types of test sentences in the two test conditions. That is, each participant was tested with the four stories provided. For both the child group and the adult control group, we had 60 test items (3 test sentences ^∗^ 20 subjects) for each type of the test sentences in each condition, and the same number of filler sentences. The number of ‘Yes’ and ‘No’ responses were counterbalanced. The two types of the test sentences were counterbalanced among the participants, and they were tested on two different sessions, with at least half a day apart. Each session consisted of two stories, with one story presenting the amount-oriented context (Condition 1) and the other one presenting the individual-oriented context (Condition 2); the ordering of the two stories was counterbalanced among the participants. Each testing session lasted about 15 min.

#### Results

**FIGURE 4:**
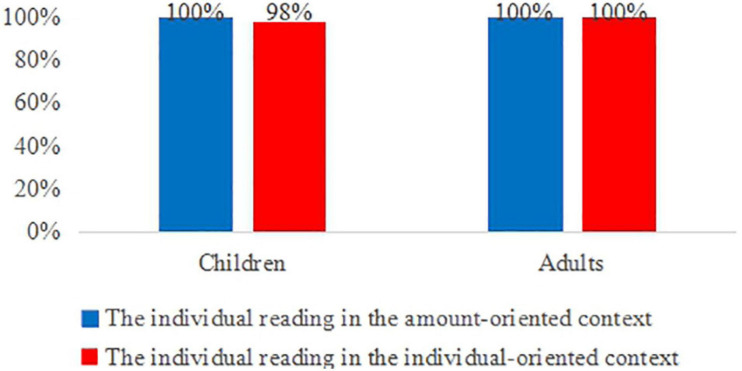
Children’s and adults’ interpretation of *duo-shao-ge* sentences in the two distinct contexts.

Let us first consider the responses to the classifier-containing *duo-shao-ge* sentences. Both children and adults accepted the test sentences over 98% of the times in both the amount-oriented context [children and adults: 100% (60/60 trials)] and in the individual-oriented context [children: 98% (59/60 trials); adults: 100% (60/60 trials)] (Figure 4). The acceptance of these test sentences indicates that the participants quantified over a cardinal scale and made the quantity judgment based on the number of individual objects in the two test conditions, as the two animals in question acted upon the same number of individual objects in our story situations (e.g., one animal ate two big strawberries, and the other ate two small strawberries). The data hence suggest that both children and adults assigned the individual-denoting reading to the *duo-shao-ge* sentences in the two distinct contexts, and the interpretation of this type of sentences is thus independent of context. This confirms our theoretical analysis of the *wh*-pronominal phrase *duo-shao-ge*-N.

**FIGURE 5:**
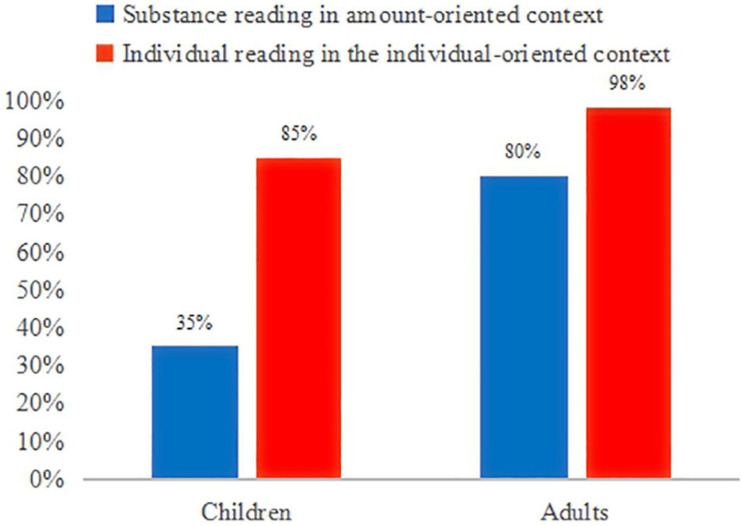
Children’s and adults’ interpretation of *duo-shao* sentences in the two distinct contexts.

The experimental data on the bare *duo-shao* sentences present a more complicated picture (see Figure 5). Consider the adults’ data first. In the individual-oriented context, they accepted the *duo-shao* sentences 98% of the times (59/60 trials). This suggests that adults quantified over the number of individual objects in the story situations and assigned the individual-denoting reading to the *duo-shao* sentences in this context. Conversely, in the amount-oriented context, adults rejected the *duo-shao* sentences 80% of the times (48/60 trials). In justifying their rejections of the puppet’s statements, they pointed out that the two animals in question acted upon uneven amounts of objects. For instance, in their justification for the rejection of sentence (14), participants pointed out that Rabbit ate the big carrots, while Horse ate the small carrots. The high percentage of the rejection rate (i.e., 80%) in the amount-oriented context hence indicates that the majority of the adults quantified over the amount of objects and assigned the substance-denoting reading to the *duo-shao* sentences in this amount-oriented context. A Wilcoxon-test shows that adults chose the individual-denoting reading for the *duo-shao* sentences in the amount-oriented context significantly less than in the individual-oriented context (20% vs. 98%, *Z* = 3.9, *p* < 0.001). Thus, we conclude that the adults made a clear distinction in their responses to the *duo-shao* sentences in these two conditions.

By examining each adult participant’s responses to the *duo-shao* sentences across the two test conditions, we found that 80% of the adults (16 out of 20 adults) exhibited both the individual-denoting and substance-denoting readings in their interpretation of the *duo-shao* sentences. They assigned the substance-denoting reading in the amount-oriented context and the individual-denoting reading in the individual-oriented context. We call this pattern of responses Pattern I: a combination of the individual-denoting and substance-denoting readings. Moreover, 20% of the adults (4 out of 20 adults) accepted the *duo-shao* sentences across the two distinct contexts and assigned exclusively the individual-denoting reading to the *duo-shao* sentences. These four adults showed a preference of the individual-denoting reading, and did not change their interpretation of this type of sentences even in the amount-oriented context. We call this Pattern II: an individual-denoting reading.

Now consider children’s responses to the *duo-shao* sentences. In the individual-oriented context, they accepted the test sentences 85% of the times (51/60 trials), assigning the individual-denoting reading to the *duo-shao* sentences in this context. In the amount-oriented context, children rejected the test sentences 35% of the times (21/60 trials) and assigned the substance-denoting reading in this context (Figure 5). Their rejections were justified by mentioning the uneven amounts of objects in the stories, just as the adults did in the same situations. This means that 65% of the times children still access the individual-denoting reading in the amount-oriented context. Moreover, a Mann-Whitney test shows that children assigned the individual-denoting to the *duo-shao* sentences in the amount-oriented context significantly more than adults did in the same context (children:65%; adults:20%; *Z* = 2.842, *p* < 0.05). Nevertheless, the children made a clear distinction in their responses to the *duo-shao* sentences in the two conditions, as they assigned the individual-denoting reading to the *duo-shao* sentences in the amount-oriented context significantly less than in the individual-oriented context (65% vs. 85%, Wilcoxon-test, *Z* = 2.0, *p* < 0.05).

Three patterns of responses are found in the children’s interpretation of the *duo-shao* sentences, including the two patterns identified in the adult group and an additional pattern. First, 20% of the children (4/20) rejected the *duo-shao* sentences in the amount-oriented context but accepted them in the individual-oriented context, exhibiting both the substance-denoting reading and the individual-denoting reading. These children behaved like the majority of the adult group, and belong to Pattern I as defined above. Second, 65% of the children (13/20) accepted the *duo-shao* sentences in the two distinct contexts, assigning only the individual-denoting reading to the sentences (Pattern II). Third, 15% of the children (3/20) rejected the *duo-shao* sentences in the two distinct contexts, and assigned exclusively the substance-denoting reading to the sentences. These children justified their rejections by pointing out the uneven “amount” of objects in the amount-oriented context (e.g., Rabbit ate the big carrots, but Horse ate the small carrots), and the different sizes of objects in the individual-oriented context (e.g., Rainbow Bird made the big flowers, but Duck made the small flowers). We call this pattern, not attested in the adult group, Pattern III: a substance-denoting reading.

**FIGURE 6:**
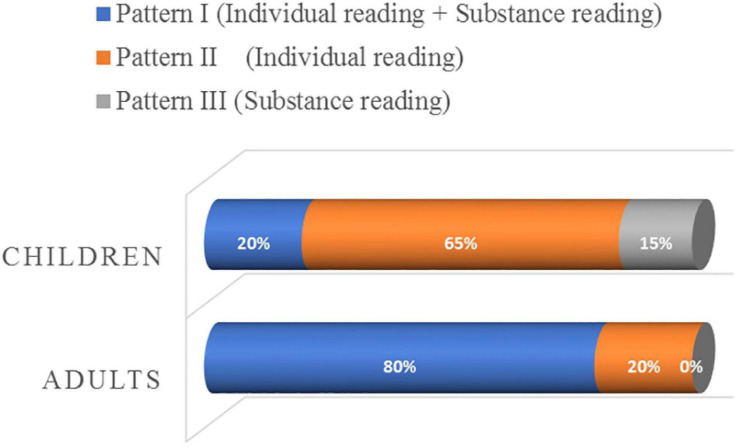
Patterns of responses in the interpretation of the *duo-shao* sentences.

The distribution of the three patterns of responses is summarized in Figure 6.

Comparing the individual data of the adult group and the child group, we can conclude that the majority of the adult group are sensitive to the distinct contexts provided, and assign the substance-denoting reading and the individual-denoting reading to the bare *duo-shao* sentences in the specific contexts. Moreover, 4-to-5-year-old Mandarin-speaking children start assigning the individual-denoting and substance-denoting readings to the bare *duo-shao* sentences, but they are still not as sensitive to the contextual information as adults are. Children showed a preference for the individual-denoting reading in their interpretation of the *duo-shao* sentences.

#### Discussion

Now we are ready to answer the two related research questions we raised for Experiment 1. Our first question was whether Mandarin-speaking children would behave like adults and allow both individual-denoting and substance-denoting readings in interpreting bare *duo-shao* sentences, but would allow only individual-denoting readings in interpreting classifier-bearing *duo-shao-ge* sentences. Our second question was whether Mandarin-speaking children know that contextual manipulation affects the interpretation of bare *duo-shao* sentences but not that of classifier-bearing *duo-shao-ge* sentences.

The experimental results allow us to give positive answers to these two questions. First, children treated the classifier-bearing *duo-shao-ge* sentences differently from the bare *duo-shao* sentences in specifying a unit of counting. They assigned exclusively the individual-denoting reading to the classifier-bearing sentences across the two distinct contexts. By contrast, they started assigning multiple readings to the bare *duo-shao* sentences, exhibiting three distinct patterns of responses in their interpretation of this type of sentences. Furthermore, even though children showed a preference for the individual-denoting reading in their interpretation of the *duo-shao* sentences in the amount-oriented context, a Wilcoxon-test shows that the percentage (65%) is still significantly lower than the percentage (100%) of the individual-denoting reading that they assigned to the *duo-shao-ge* sentences in the same amount-oriented context (*Z* = 2.646, *p* < 0.01). The assignment of the multiple readings assigned to the bare *duo-shao* sentences indicate that multiple scales of measurement are adopted in the interpretation of this type of sentences, due to the lack of a linguistic element encoding the portioning-out function. On the other hand, the assignment of the sole individual-denoting reading to the *duo-shao-ge* sentences indicate that only a cardinal scale is adopted, due to the *portioning-out* function of *ge*. Therefore, we conclude that Mandarin-speaking children are well aware of the portioning-out function of Mandarin classifiers, and they are sensitive to the interpretive differences caused by the presence/absence of a classifier in their interpretation of these Mandarin *wh*-pronominal phrases.

Second, children also showed that contextual manipulation (amount-oriented context vs. individual-oriented context) affected their interpretation of the bare *duo-shao* sentences, but not the classifier-bearing *duo-shao-ge* sentences. In interpreting the *duo-shao-ge* sentences, they behaved like adults and assigned rigidly the individual-denoting reading in both the amount-oriented and individual-oriented contexts. On the other hand, children started assigning both the individual-denoting reading and the substance-denoting reading to the *duo-shao* sentences in the appropriate contexts, even though they were not yet sensitive to the contextual information as adults were.

This brings us to an issue raised by one reviewer, namely, why few adults (4 out of 20) and more than half of the children (65%) assigned the individual-denoting reading to the bare *duo-shao* sentences in the amount-oriented context. Although we do not have an explicit answer to this question, we still think these ‘participants’ behavior fits with our proposal. According to our account, in fact, *duo-shao* is ambiguous between the individual-denoting reading and the substance-denoting reading. So the assignment of the alternative readings largely depends on how participants are sensitive to the specific contexts we designed. Even though we aimed to trigger the substance-denoting reading in the amount-oriented context and the individual-denoting reading in the individual-oriented context, the percentage of either interpretation is never at ceiling. The sentence remains ambiguous and preference for one particular reading can be hard to override. The absolute accuracy only applies to those sentences that are not ambiguous at all, like the *duo-shao-ge* sentences as show above.

This concludes our report of Experiment 1.

### Experiment 2

Let us now turn to our second experiment, designed to investigate whether and at what age children are able to apply the individuation function of the classifier *ge* in interpreting the *wh*-*wh*-pronominal phrase *duo-shao-ge*-N. Such function determines that phrases containing *duo-shao-ge* can only refer to whole objects (and not their parts).

#### Test Sentences

A typical test sentence is shown in (19). In this sentence, the *wh*-pronominal phrase *duo-shao-ge*-N is contained in the same conditional structure we used in Experiment 1.

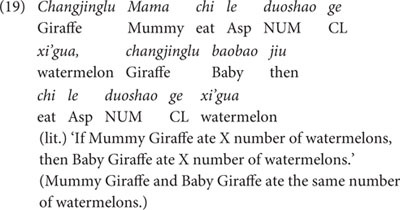

Due to the individuation function of the classifier *ge*, the phrase *duo-shao-ge-xi’gua* in (19) has to denote individual watermelons, and cannot denote non-individuals such as slices of watermelons. Therefore, sentence (19) can only receive an individual-denoting reading: ‘Mummy Giraffe and Baby Giraffe ate the same number of individual watermelons.’

#### Participants and Experimental Method

Two groups of 20 children participated in this experiment. The first group ranged in age from 4;3.11 to 5;5.6 (mean age 5;1.3); the second group ranged in age from 6;4.15 to 6;9.25 (mean age 6;7.23). We call these two groups of children ‘5-year-old group’ and ‘6-year-old group,’ respectively. We also included a control group of twenty adults, with a mean age of 20 years. The child and adult participants are not the same ones in Experiment 1.

We adopted the same experimental method used in Experiment 1, namely, the Truth Value Judgment Task. Like we did in Experiment 1, we tested the child participants individually, and tested the adult participants in a group. There was a practice trial to familiarize the participants with the task, and only those participants who correctly responded in the practice trial proceeded to the test session.

#### Test Conditions and Materials

**FIGURE 7:**
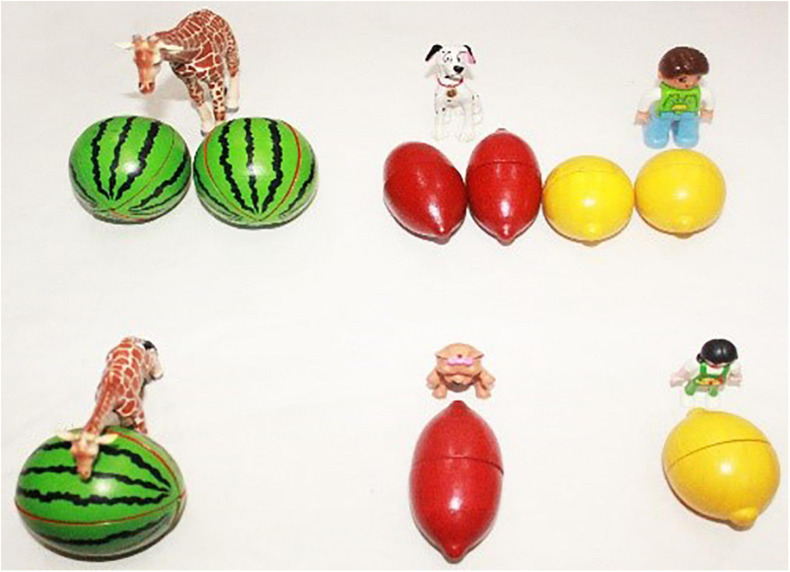
Last scene of the story of the Whole Object Condition.

There were two test conditions, including the Whole Object Condition and the Partial Object Condition, and these two test conditions corresponded to two events of a story. In the Whole Object Condition, three pairs of characters, i.e., Mummy Giraffe and Baby Giraffe, Mummy Dog and Baby Dog, and a boy and a girl, went to buy food for a picnic. While the first member of each pair bought one food item, the second one bought two food items of the same kind and size. The English translation of the story script is shown in (20). The last scenario of the story is shown in Figure 7.

(20) A boy and a girl planned to have a picnic with their animal friends: Mummy Giraffe and Baby Giraffe, Mummy Dog and Baby Dog. They went to a supermarket to buy food. Mummy Giraffe bought two watermelons while Baby Giraffe bought one watermelon; Mummy Dog bought two sweet potatoes while Baby Dog bought one sweet potato; the boy bought two lemons while the girl bought one lemon.

Right after the narration of this part of the story, the puppet was invited to say what had happened in the story. Then the puppet replied by uttering three test sentences containing *duo-shao-ge*. An example is given in (21), which compares the number of watermelons bought by Mummy Giraffe and Baby Giraffe. The other two sentences, which we omit here, are of the same structure, comparing the number of other vegetables bought by other two pairs of characters (Mummy Dog and Baby Dog buying sweat potatoes, the boy and the girl buying lemons). The participants were asked to judge separately whether the puppet’s statements were true or false.

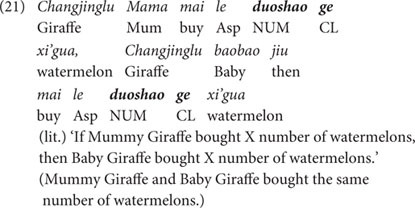

On the individual-denoting reading (‘Mummy Giraffe and Baby Giraffe bought the same number of watermelons’), the example sentence (21) is a false description of the story and should be rejected, because Mummy Giraffe bought two watermelons while Baby Giraffe bought only one watermelon in the story.

**FIGURE 8:**
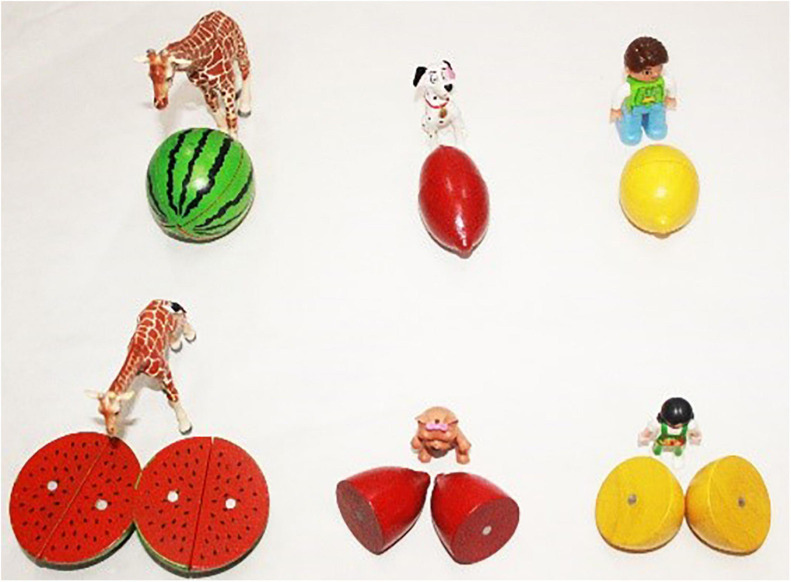
Last scene of the story of the Partial Object Condition.

In the Partial Object Condition, the same three pairs of characters each ate one food item, but they ate the food in two different ways: while one member of the pair ate his/her food with one gulp, the other one cut the food into two pieces and ate the two pieces separately. The English translation of this part of the story is given in (22). The last scenario of the story is shown in Figure 8.

(22) The boy, the girl and their animal friends got tired, so they took a nap. When they were fast asleep, a mouse came to steal their food. The mouse stole a watermelon from Mummy Giraffe, a sweet potato from Mummy Dog, and a lemon from the boy. After a while, the boy, the girl and their animal friends woke up, and found one of their food items had been stolen. So they started their picnic immediately. The boy, Mummy Giraffe and Mummy Dog were very hungry, and ate their food in one gulp. The girl, Baby Giraffe and Baby Dog cut their food in half, and then each of them ate the two pieces one by one.

After this part of the story, the puppet was invited again to state what had happened. The puppet produced another set of three *duo-shao-ge* sentences. An example is given in (23), which compares the number of watermelons eaten by Mummy Giraffe and Baby Giraffe. The other two sentences, which we omit here due to the limit of space, are of the same structure comparing the number of vegetables eaten by two other pairs of characters.

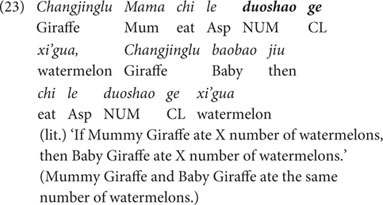

Sentence (23) conveys the individual-denoting reading ‘Mummy Giraffe and Baby Giraffe ate the same number of watermelons’ and it is a true description of the story: the two halves eaten by Baby Giraffe came from a whole watermelon, and hence Baby Giraffe ate the same number of watermelons as Mummy Giraffe did, who did not cut her watermelon and ate it in one gulp. Therefore, adults were expected to accept the test sentences in this test condition.

As for children, however, considering the possible delay of the individuation function of Mandarin classifiers (cf. Huang, 2009; Huang and Lee, 2009; Duan, 2011), we predict that young children might reject the three test sentences in the Partial Object Condition. To exemplify with sentence (23), if young children are not yet aware of the individuation function of the classifier *ge*, they would then quantify over discrete entities and count two halves of the watermelon eaten by Baby Giraffe as ‘two watermelons.’ Therefore, for young children Baby Giraffe did not eat the same number of watermelons as Mummy Giraffe did, who ate one whole watermelon. This would lead to their rejection of the target sentence, which states that the two characters ate the same number of watermelons.

In addition, the puppet produced three simple sentences as shown in (24)–(26) for additional information on children’s acquisition of the individuation function of *ge*.

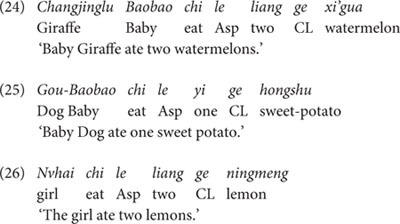

The three sentences comment upon the number of food items eaten by the characters in the story who cut their food in half. For the reason explained above, if children acquire the individuation function of the classifier *ge*, they would reject sentences (24) and (26), which state that the animal characters ate two vegetables, and accept sentence (25), which states that the animal character ate one vegetable. Otherwise, they would accept (24) and (26), but reject (25). The filler sentences give us an additional source to look into the individuation associated with *duo-shao-ge* phrases.

#### Results

In the Whole Object Condition, both adults and children correctly rejected the test sentences 100% of the times (60/60 trials). They justified their rejections by mentioning the uneven number of food items that bought by the two characters in each test sentence. For example, a typical justification for the rejection of sentence in (21) is that while Mummy Giraffe bought two watermelons, Baby Giraffe bought only one watermelon.

In the Partial Object Condition, adults accepted the test sentences 95% of the times (57/60 trials). The high acceptance of the test sentences in this condition indicates that adults considered two halves as one individual object, thus assigning the individuation function to the individual classifier *ge*. Children exhibited a developmental pattern in their responses to the test sentences in this condition. In particular, the group of 5-year-old children accepted the test sentences only 35% of the times (21/60 trials), but the percentage increased to 90% of the times (54/60 trials) in the 6-year-old-group. A Mann-Whitney test shows that the 6-year-old children accepted the test sentences significantly more often than the 5-year-old children (*Z* = 3.547, *p* < 0.01), but there is no significant difference between the 6-year-old children and adults (*Z* = 0.593, *p* > 0.05). This result shows that children do not acquire the individuation function of the individual classifier *ge* until they reach the age of 6. This generalization is confirmed by children’s justifications. For instance, when rejecting sentence (23), one child stated Baby Giraffe had eaten ‘two watermelons,’ as shown in (27).

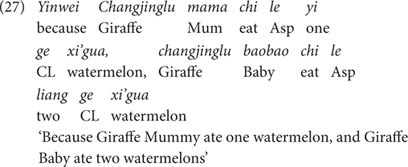

**FIGURE 9:**
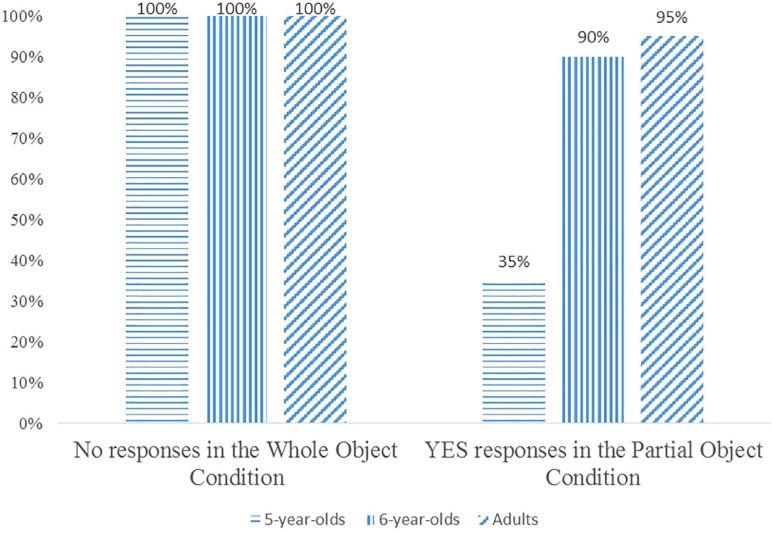
Children’s and adults’ responses in the Whole Object Condition and the Partial Object Condition.

Clearly, the child used the individual classifier phrase *liang ge xi’gua* ‘two watermelons’ to refer to two halves of a whole watermelon eaten by Giraffe Baby. Thus, younger children who did not acquire the individuation function of *ge* quantified over discrete entities, and rejected the test sentences in the Partial Object Condition just as they did in the Whole Object Condition. The experimental data are summarized in Figure 9 below.

Further confirmation comes from children’s responses to the sentences (24)–(26). Two kinds of responses are observed. First, the children who rejected the test sentences in the Partial Object Condition accepted the sentences (24) and (26), and rejected the sentence (25). These children did not acquire the individuation function of *ge*, allowing *duo-shao-ge* to quantify over discrete entities and counting two halves of a food item as ‘two food items.’ 65% of the children (13 out of 20) from the 5-year-old group exhibited this pattern of response. Second, those who correctly accepted the test sentences in the Partial Object Condition rejected the sentences (24) and (26) but accepted the filler sentence (25) as adults did. These children exhibited answers underlining an adult-like grammar in both kinds of sentences, and assigned the individuation function to *ge*. Hence, they considered two halves as one single individual object in their comprehension of the *duo-shao-ge* phrases. 90% of the children (18 out of 20) in the 6-year-old group displayed this pattern of response.

To wrap up, Experiment 2 shows that the individuation function of Mandarin classifiers in *duo-shao-ge* is delayed in Mandarin-speaking children. Children do not acquire this function until they reach the age of 6. These results are consistent with the findings from previous studies on the acquisition of the individuation function of Mandarin classifiers (Huang, 2009; Huang and Lee, 2009; Duan, 2011).

## General Discussion and Conclusion

In the present study, we conducted two experiments to investigate the portioning-out and individuation functions in the minimal pairs of *wh*-pronominal phrases with and without the classifier *ge*, i.e., *duo-shao*-N and *duo-shao-ge*-N. In Experiment 1, we found that 5-year-old Mandarin-speaking children were sensitive to the interpretive differences in portioning-out between these two *wh*-pronominal phrases. They assigned the individual-denoting and substance-denoting readings to *duo-shao*-N, but only the individual-denoting reading to *duo-shao-ge*-N. This indicates children’s awareness of the portioning-out function associated with the classifier *ge*. In Experiment 2, we found that Mandarin-speaking children quantified over partial entities rather than individual objects in their comprehension of *duo-shao-ge*-N before they reached 6 years. We attribute this kind of non-adult responses to the delay of the individuation function of classifiers. Taken together, our experimental data show that Mandarin-speaking children, like adults, allow both count and mass readings in their interpretation of the bare *wh*-pronominal phrase *duo-shao*-N, and the portioning-out and individuation functions of the individual classifier *ge* associated with *duo-shao-ge*-N develop independently in the course of language development, with the portioning-out function taking precedence over the individuation function.

Based on our experimental findings, the present study can help adjudicate the main alternative accounts of the Mandarin count-mass issue, as reviewed in Section “Portioning-Out and Individuation in Mandarin.” First of all, our experimental data give support to the view that individuation is encoded in classifiers rather than in nouns (Hansen, 1983; Bach, 1989; Graham, 1989; Krifka, 1995; Chierchia, 1998; Borer, 2005; Huang, 2009; Huang and Lee, 2009; Rothstein, 2010; Pelletier, 2012). As clearly shown in our Experiment 2, individuation is unambiguously specified with the presence of the individual classifier *ge* in the sentences containing *duo-shao-ge*, but not in the sentences containing the bare *wh*-pronoun *duo-shao*. Such a contrast between the minimal pair *duo-shao* and *duo-shao-ge* allows us to see that bare elements like *duo-shao*-N phrases do not specify a fixed count or mass interpretation, and it is classifiers that play the decisive role of encoding individuation in Mandarin.

This brings us to our comments on the lexico-syntactic account proposed by Cheng and Sybesma (1998), which claim that individuation is specified in nouns rather than in classifiers (see section “Portioning-Out and Individuation in Mandarin”). This account would predict that only individual-denoting readings are available for the nouns used in our experiment (i.e., *nangua* ‘pumpkin,’ *huluobo* ‘carrot’ and *baicai* ‘cabbage’). According to this account, these nouns would be classified as count nouns, as they “present themselves naturally in discrete, countable units,” and the function of individual classifiers is merely to “name” the natural unit of counting and make the semantic partitioning of the count nouns syntactically visible. In other words, contra our experimental findings, the lexico-syntactic account would not expect an interpretive difference between the nouns that co-occur with *duo-shao* and the nouns that co-occur with *duo-shao-ge*; and this account would not expect the multiple readings of the nouns co-occurring with *duo-shao* either. Therefore, the lexico-syntactic account proposed by Cheng and Sybesma cannot explain our experimental data, and the present study poses a challenge to this account.

Furthermore, both Borer (2005) and Pelletier (2012) hold that individuation is specified by classifiers, but the present study offers empirical evidence showing that Pelletier’s account fares better than Borer’s account in their characterization of bare nouns in Mandarin. As reviewed in Section “Portioning-Out and Individuation in Mandarin,” while Borer argues that bare nouns are mass by default, Pelletier holds that both count and mass interpretations are available for bare nouns. In our Experiment 1, both count and mass readings are attested in Mandarin-speaking children’s and adults’ interpretation of the sentences containing *duo-shao*.

In a word, among the three accounts on the Mandarin count-mass issue, Pelletier (2012) is the one that is consistent with our experimental data. All the main ideas of this account (i.e., individuation is specified by classifiers, and Mandarin bare nouns allow both count and mass interpretations) are empirically supported in our experiments. In the literature, a similar discussion on the interpretation of bare nouns can be found in Lin and Schaeffer (2018). In this study, 2-to-5 Mandarin-speaking children and adults are reported to assign both count and mass readings to three types of bare nouns, including count nouns (e.g., *qiu* ‘ball’), mass nouns (e.g., *mianfen* ‘flour’), flexible nouns (e.g., *shengzi* ‘string’), even though various preferences are identified due to the factor of linguistic experience. However, this study only tested Mandarin-speaking children’s and adults’ interpretation of bare nouns in their experiments. In our experiments, we tested the interpretation of both bare nouns and classifier-bearing phrases. In this regard, we provide new and more convincing data for the study of the Mandarin count-mass issue.

Our experimental data are consistent with the findings from Huang (2009), Huang and Lee (2009), and Duan (2011), which report that the portioning-out function of Mandarin classifiers is acquired earlier than their individuation function (see section “Portioning-Out and Individuation in Mandarin *wh*-Pronominal Phrases”). From a cross-linguistic perspective, our experimental data are also consistent with the findings on the asymmetric acquisition of portioning-out and individuation in the interpretation of English plural morphology (see section “Introduction”). Thus, in both Mandarin and English, the portioning-out function emerge earlier than the individuation function in the course of language development. The asymmetric development of these two functions suggests, first of all, that the portioning-out function is more fundamental than the individuation function (Au Yeung, 2005), considering the assumption that core linguistic properties are part of the initial state of our grammar and occur early in the course of language development (Crain, 2012). This generalization is also compatible with the observation that the portioning-out function is the basic function of all Mandarin classifiers, while the individuation function is a special function encoded only in certain classifiers such as individual classifiers and collective classifiers (see section “Portioning-Out and Individuation in Mandarin *wh*-Pronominal Phrases”). Moreover, the cross-linguistic parallel suggests that, languages may differ in their ways of encoding portioning-out and individuation by using typological distinct formal categories (e.g., the plural morphology and count determiner in English, individual classifiers in Mandarin), but what these formal categories convey are similar in semantic functions.

Before we conclude the paper, we consider a remaining issue raised by the reviewer about children’s non-adult responses in Experiment 2, i.e., young children’s quantifying over partial objects and counting two halves of a watermelon as ‘two watermelons.’ We attribute the lack of the ‘wholeness requirement’ to the delay of the individuation function of the individual classifier *ge* in children’s early grammar. However, the reviewer asked how we can exclude the possibility that it is actually the delay of “knowledge of the world”: young children do not know how complete an object needs to be for it to be considered an individual object.

We do not have independent data to rule out this possibility. However, we can do so by resorting to some experimental findings as reported by Brooks et al. (2011). As we introduced in Section “Introduction,” this study found that 4-year-old English-speaking children treated pieces of broken things as units of counting when interpreting count quantifiers like *more*, *every*, *both*, and when labeling sets using plural morphology (Experiment 1). Furthermore, this study also found that when two familiar objects (e.g., two cups) were glued together, 4-year-old children counted the glued things as two rather than one (Experiment 2). Moreover, 4-year-old children did not include parts of objects with specific names (e.g., wheels of a bicycle) in their counting (Experiment 3) (see Srinivasan et al., 2013 for similar findings). Clearly, these experimental data indicate that 4-year-old children knew well what constitutes an individual object. Therefore, we believe that 4-year-old English-speaking children accepted broken objects as units of counting, not because they did not know how complete an object should be in order to be called an individual object, but because they had not acquired the individuation function of those count quantifiers. Adopting the arguments of Brooks et al. (2011) to explain our Mandarin data, we hold that Mandarin children’s non-adult behavior is not due to the lack of the real world knowledge about what constitutes an individual object. Rather, we attribute the non-adult behavior to the delay of the individuation function of *ge*, as we argue throughout the paper.

Furthermore, Brooks et al. (2011) propose that the learning of names for parts of objects (e.g., *wheel*) and unitizers like *chunk*, *bit*, *slice*, *portion*, and *piece* (e.g., piece of a shoe) could help English children attain the adult grammar. The acquisition of these expressions could indicate to children that pieces of things are labeled differently from whole things: parts of shoes should be counted as *pieces of shoes* rather than as *shoes* (see also Srinivasan et al., 2013). We think the same acquisition strategy may be applied by Mandarin children. Through the acquisition of classifiers such as *kuai* ‘chunk’ ‘piece’ and names for parts of objects, Mandarin children gradually understand that partial objects should be referred to by non-individual classifier phrases or specific nouns, restricting individual classifier phrases to refer to individual objects. Of course, more research needs to be done to explore these issues, and we leave it for future endeavors.

Now we can conclude the paper. In line with the previous research, the present study contributes new data to support the view that portioning-out and individuation are encoded in classifiers rather than in nouns, and bare linguistic expressions are underspecified in portioning-out and individuation.

## Data Availability Statement

The original contributions presented in the study are included in the article/supplementary material, further inquiries can be directed to the corresponding author/s.

## Ethics Statement

The studies involving human participants were reviewed and approved by Human Research Ethics Committee, School of Foreign Languages, Soochow University, China. Written informed consent to participate in this study was provided by the participants’ legal guardian/next of kin.

## Author Contributions

AH designed the experiments, collected the data, and drafted the whole article. F-AU and LM discussed and edited the article. All authors contributed to the article and approved the submitted version.

## Conflict of Interest

The authors declare that the research was conducted in the absence of any commercial or financial relationships that could be construed as a potential conflict of interest.

## Footnotes

1 We thank a reviewer for pointing out and suggesting these two related semantic dimensions in our characterization of the portioning-out function.

2 This claim does not apply to those measure classifiers which specify a specific weight, such as the measure classifier *jin* ‘500 g.’ We would thank a reviewer for pointing this out for us.

3 *Duo-shao*-N and *duo-shao-ge*-N, like other *wh*-pronominal phrases in Mandarin, can be used in interrogative or non-interrogative sentences. Since our experiments used only a non-interrogative form of these two *wh*-pronominal phrases, we do not discuss about their interrogative uses here. The *wh*-pronoun *duo-shao* can be followed with other types of classifiers but we mainly consider the situation in which *duo-shao* is used with the individual classifier *ge*, as this is enough for us to investigate portioning-out and individuation in Mandarin.
